# Genetic etiology and clinical challenges of phenylketonuria

**DOI:** 10.1186/s40246-022-00398-9

**Published:** 2022-07-19

**Authors:** Nasser A. Elhawary, Imad A. AlJahdali, Iman S. Abumansour, Ezzeldin N. Elhawary, Nagwa Gaboon, Mohammed Dandini, Abdulelah Madkhali, Wafaa Alosaimi, Abdulmajeed Alzahrani, Fawzia Aljohani, Ehab M. Melibary, Osama A. Kensara

**Affiliations:** 1 Department of Medical Genetics, College of Medicine, Umm Al-Qura University, P.O. Box 57543, Mecca, 21955 Saudi Arabia; 2 Department of Community Medicine, College of Medicine, Umm Al-Qura University, P.O. Box 57543, Mecca, 21955 Saudi Arabia; 3 Faculty of Medicine, MS Genomic Medicine Program, University of Southampton, Southampton General Hospital, Southampton, UK; 4 Department of Clinical Genetics, Faculty of Medicine, Ain Shams University, Cairo, Egypt; 5 Department of Laboratory and Blood Bank, Maternity and Children Hospital, Mecca, Saudi Arabia; 6 Department of Pathology and Laboratory Medicine, King Abdulaziz Medical City, Ministry of National Guard Health Affairs, Riyadh, Saudi Arabia; 7 Department of Hematology, Maternity and Children Hospital, Mecca, Saudi Arabia; 8 Department of Laboratory and Blood Bank at Maternity and Children Hospital, Mecca, Saudi Arabia; 9 Department of Pediatric Clinics, Maternity and Children Hospital, King Salman Medical City, Madinah, Saudi Arabia; 10 Department of Clinical Nutrition, Faculty of Applied Medical Sciences, Umm Al-Qura University, Jeddah, Saudi Arabia; 11 Department of Biochemistry, Batterjee Medical College, Jeddah, Saudi Arabia

**Keywords:** Phenylketonuria, Phenylalanine hydroxylase, Tetrahydrobiopterin, Epidemiology, Genetic etiology, Pathophysiology, PKU management

## Abstract

This review discusses the epidemiology, pathophysiology, genetic etiology, and management of phenylketonuria (PKU). PKU, an autosomal recessive disease, is an inborn error of phenylalanine (Phe) metabolism caused by pathogenic variants in the phenylalanine hydroxylase (*PAH*) gene. The prevalence of PKU varies widely among ethnicities and geographic regions, affecting approximately 1 in 24,000 individuals worldwide. Deficiency in the PAH enzyme or, in rare cases, the cofactor tetrahydrobiopterin results in high blood Phe concentrations, causing brain dysfunction. Untreated PKU, also known as PAH deficiency, results in severe and irreversible intellectual disability, epilepsy, behavioral disorders, and clinical features such as acquired microcephaly, seizures, psychological signs, and generalized hypopigmentation of skin (including hair and eyes). Severe phenotypes are classic PKU, and less severe forms of PAH deficiency are moderate PKU, mild PKU, mild hyperphenylalaninaemia (HPA), or benign HPA. Early diagnosis and intervention must start shortly after birth to prevent major cognitive and neurological effects. Dietary treatment, including natural protein restriction and Phe-free supplements, must be used to maintain blood Phe concentrations of 120–360 μmol/L throughout the life span. Additional treatments include the casein glycomacropeptide (GMP), which contains very limited aromatic amino acids and may improve immunological function, and large neutral amino acid (LNAA) supplementation to prevent plasma Phe transport into the brain. The synthetic BH4 analog, sapropterin hydrochloride (i.e., Kuvan®, BioMarin), is another potential treatment that activates residual PAH, thus decreasing Phe concentrations in the blood of PKU patients. Moreover, daily subcutaneous injection of pegylated Phe ammonia-lyase (i.e., pegvaliase; PALYNZIQ®, BioMarin) has promised gene therapy in recent clinical trials, and mRNA approaches are also being studied.

## Background

Phenylketonuria (PKU, MIM 261,600) is a deficiency in the hepatic enzyme phenylalanine hydroxylase (PAH; EC 1.14.16.1; OMIM 612,349) that occurs in approximately 1 in 24,000 people, with an estimated 450,000 individuals affected worldwide [1]. Also known as PAH deficiency, PKU is an inborn error of phenylalanine (Phe) metabolism caused by pathogenic variants in the *PAH* gene. It is inherited in an autosomal recessive pattern. The PAH enzyme, expressed predominantly in the liver (but also kidney and pancreas), is responsible for the conversion of Phe to tyrosine (Tyr) in a reaction that requires the co-substrate tetrahydrobiopterin (BH4). Of note, BH4 and DNAJC12 can also act as chaperones to facilitate the proper folding of the PAH monomer [2–5]. Thus, a small number of cases of hyperphenylalaninaemia (HPA) are caused by defects in BH4 metabolism or pathogenetic variants in the *DNAJC12* gene. HPA is the core biochemical abnormality of PKU, in which blood Phe concentrations exceed the normal range of 35–120 μmol/L.

In untreated patients with PKU, blood Phe concentrations significantly increase, resulting in phenylpyruvic acid excreted in the urine. Conversely, Tyr concentrations are usually somewhat low. Untreated patients are characterized by severe intellectual disability, epilepsy, seizures, psychological behaviors, acquired microcephaly, generalized skin hypopigmentation, and a musty sweat odor [6–9]. Generally, individuals with severe phenotypes have classic PKU, and those with less severe PAH deficiency have moderate PKU, mild PKU, mild HPA, or benign HPA. However, classification should be considered carefully, as patients can be diagnosed before reaching high levels of Phe [10, 11]. According to the first European classification guidelines for PAH deficiency, the disorder ranges from mild HPA (Phe concentrations of 120–360 μmol/L; no treatment necessary) to PKU (Phe concentrations > 360 μmol/L), which can be further categorized as BH4-responsive PKU or BH4-non-responsive PKU [12]. PKU was the first disorder to benefit from newborn screenings and can be manageable if detected early in life. Current treatment options, which aim to reduce Phe blood concentration, include following a low-Phe diet and trying new drug mechanisms [13, 14].

The *PAH* gene, mapped to chromosome 12 (12q22–q24.2), is 90 kb in length with 13 exons [7]. PKU is genetically heterogeneous, with more than 1,000 *PAH* variants reported in individuals with PKU worldwide [1, 15]. These PAH variants are cataloged in the locus-specific databases PAHvdb and BioPKU (http://www.biopku.org).

This review discusses the epidemiology, pathophysiology, genetic etiology, and clinical management of PKU. We highlight phenotype prediction of individuals with various PAH variants and current obstacles in PKU management.

### The early history of PKU

PKU was first described in 1934 by Ivar Asbjörn Følling, who used ferric chloride to detect phenyl pyruvic acid in the urine of two Norwegian siblings with intellectual disabilities. Følling concluded that this secondary metabolite was derived from dietary phenylalanine, and later the condition was lately named [16]. PKU was first treated through dietary control in the 1950s, and then, in 1963, population-based newborn screening using dried blood spot (DBS) testing was introduced to assess Phe concentration [17], enabling early diagnosis and initiation of treatment.

### Epidemiology

**Fig. 1:**
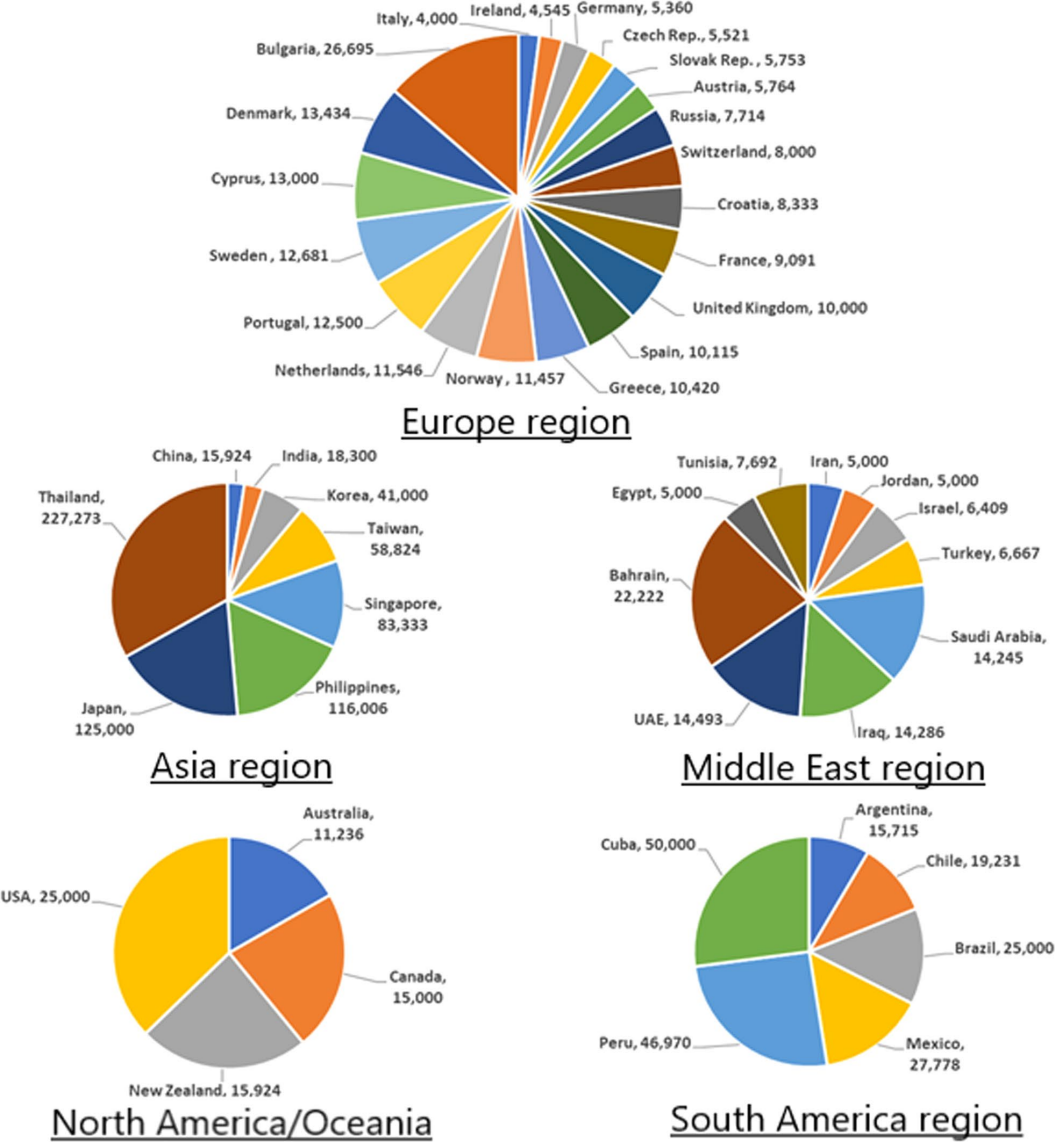
Prevalence of PKU in five world regions (prevalence, 1:*X*)

The prevalence of PKU varies widely among ethnicities and geographic regions. Worldwide, PKU has an estimated prevalence of 1 in 23,930 live births and affects about 0.45 million individuals, of whom at least two-thirds require treatment [1]. In terms of ethnicity, prevalence is generally highest in White or East Asian populations (1 in 10,000–15,000 live births) [18]. In terms of geography, it is generally lowest in Asian countries, except for China, and highest in European and Middle Eastern countries (Fig. 1).

Some of lowest PKU prevalence in Asia has been reported in Thailand (1:227,273) [19], Japan (1:125,000) [20], Philippines (1:116,006) [21], and Singapore (1:83,333) [22]; the outlier is China, where the prevalence (1:15,924) [23] is more comparable to that in Europe (Fig. 1). In the Middle East, some of the highest prevalence is in Egypt [24], Iran [25], and Jordan [26], where approximately 1:5,000 newborns are affected. Other high prevalence of PKU is 1:6,409, 1:6,667, 1:14,245, 1:14.286, 1:14,493, and 1:22,222 in Israel [1], Turkey [27], Saudi Arabia [28], Iraq [29], the United Arab Emirates [30], and Bahrain [31], respectively. The extremely high prevalence in some populations/regions may be attributed to high rates of consanguineous marriage and genetic drift/migration, especially in Arab Gulf countries, the Middle East, and the Orient [20, 32–39]. The prevalence in South America varies from 1:25,000 to 50,000 live births, with a lower prevalence in the northern than in the southern part of the continent [40].

### Pathophysiology and mental retardation

**Fig. 2:**
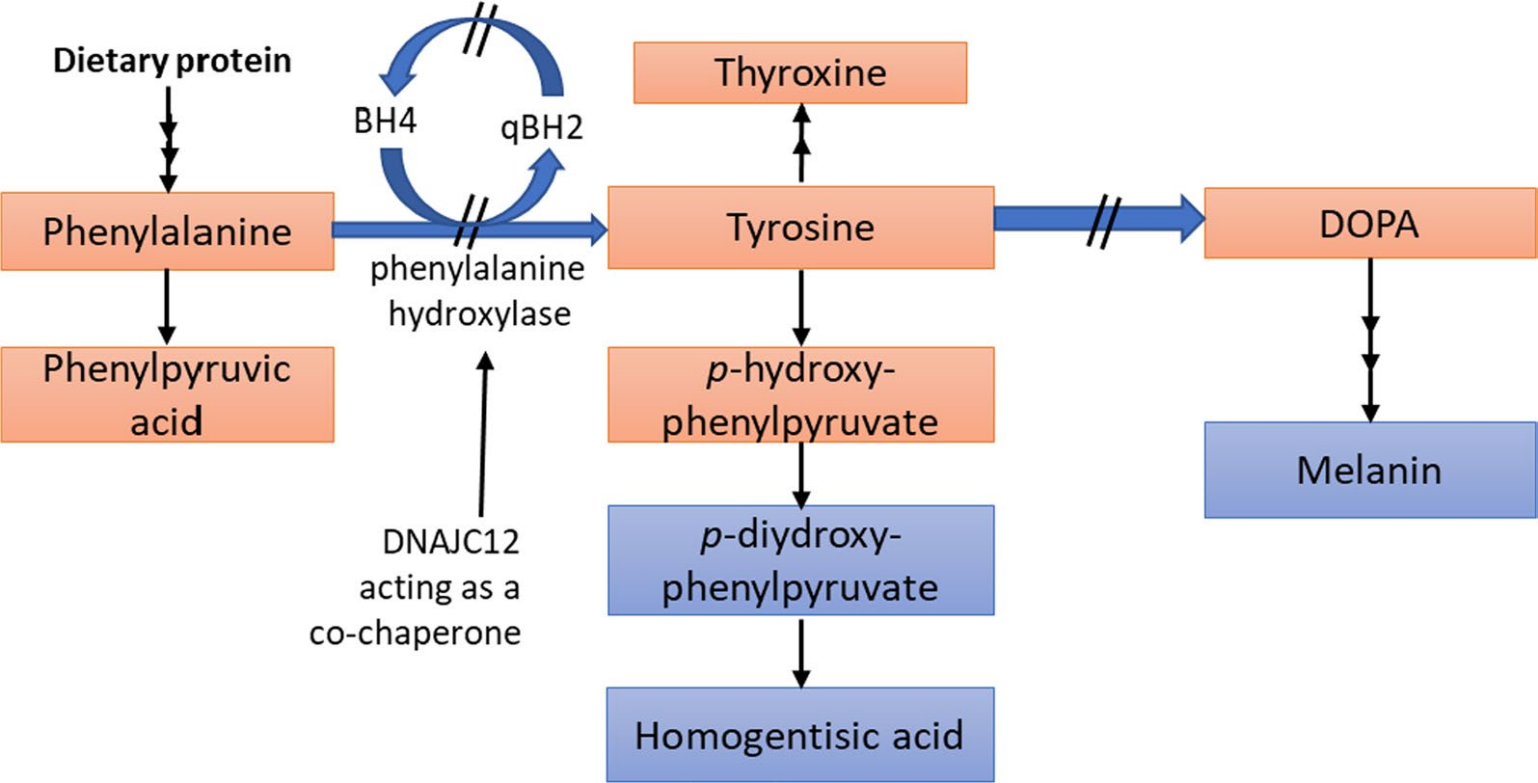
Phenylalanine metabolism in PKU. Phenylalanine hydroxylase (PAH) catalyzes the hydroxylation of L-phenylalanine to L-tyrosine

PAH is a tetrameric, iron-containing monooxygenase enzyme that catalyzes the irreversible hydroxylation of Phe to form tyrosine (Tyr) (Fig. 2). The hydroxylation of Phe requires BH4 as a cofactor in a rate-limiting step. BH4 is synthesized from guanosine triphosphate (GTP) in several tissues, including the liver, but is also recycled after Phe hydroxylation through enzymatically catalyzed reduction [41]. During hydroxylation of Phe into Tyr, BH4 is transformed to the oxidized form of quinonoid dihydrobiopterin (qBH2) [7]. The exact amount of Phe needed for net protein metabolism is still unknown. It has been reported that 10–20% of typical dietary Phe intake is utilized during normal protein turnover, and the remainder is converted into Tyr by the PAH enzyme [10].

PAH deficiency leads to the Phe accumulation in all body tissues (including the blood) and a relative tyrosine deficiency. Untreated PKU is associated with chronic HPA, microcephaly, mental retardation, epilepsy, hypopigmentation, growth delay, and eczema. Diminished BH4 levels in the prefrontal cortex also play a central role in cognitive dysfunction in PKU [42]. Inherited BH4 insufficiency is the cause of HPA in 2% of cases detected through newborn screening, requiring BH4 administration and dietary Phe restriction as a treatment course.

Because of insufficiency of other large amino acids caused by large neutral amino acid (LNAA) transporter type 1 (LAT1) competition, PKU patients also experience neurotoxic effects of Phe accumulation in the brain, in cerebral proteins, and the synthesis of neurotransmitters [43]. Blood Tyr concentrations are also reduced in patients with PKU, though hypothyroxinemia is not typically severe because of dietary Tyr intake. In patients with HPA, the deamination of excessive Phe forms phenyl lactic and phenyl pyruvic acids, which can be detected in the urine by a ferric chloride reagent. Tyr plays a major role in neurotransmitter synthesis [43], and evidence suggests that the reduction of Tyr and tryptophan (Trp) in the brain may be associated with attention-deficit hyperactivity disorder (ADHD) and HPA [44, 45]. Moreover, deficiency in the co-chaperone *DNAJC12* was recognized as a cause of inherited HPA with symptoms like BH4 deficiency [46]. *DNAJC12* helps control the folding, degradation, and translocation of hydroxylases so that a *DNAJC12* deficiency could lead to inappropriate folding of PAH [46].

Metabolic pathways include:

- The production of neurotransmitters (dopamine, adrenaline, and norepinephrine),
- Conversion to thyroxine in the thyroid gland and melanin in melanocytes, and
- Complete catabolism acetoacetate and fumarate as fuel.

### Mechanism of neuropathy

HPA causes neuronal dendritic outgrowth and synaptic connectivity disturbances in vitro [47, 48] and in vivo animal experiments [49]. In PKU patients, some of the most severe manifestations (i.e., intellectual disability and epilepsy) have a gray matter component; however, only a few studies have reported the abnormal gray matter, with most studies focusing on white matter abnormalities [50, 51]. HPA may alter the phenotypes of oligodendrocytes from myelinating to non-myelinating, but cultured oligodendrocytes from rats with HPA can lay down normal myelin sheaths [52]. Phe may impair the synthesis of cholesterol through inhibiting 3-hydroxy-3-methylglutaryl-CoA reductase activity [53] or other brain lipids, thereby interfering with myelin production. However, the precise molecular mechanisms underlying the white (and gray) matter disturbances associated with elevated brain Phe concentrations remain unknown.

### Large neutral amino acid deficiency

In HPA, Phe-mediated competition for binding to an amino acid transporter LAT1 (also known as SLCA7A5) may impair the movement of aromatic acids (i.e., Phe, Tyr, and Trp) and other LNAAs (e.g., leucine, isoleucine, valine, methionine, threonine, and histidine) into the brain across the blood–brain barrier through sodium-independent transfer [54, 55]. The resulting deficiencies are responsible for the decreased synthesis of cerebral proteins in adults with PKU [54] and contribute to neurotransmitter deficiencies in the brain [56]. The separate sodium-dependent amino acid transporters can pump amino acids outside the brain back into circulation and may modulate any disturbances of amino acid homeostasis in the brain [57]. Thus, oral supplements of LNAAs other than Phe can be useful as a treatment to correct cerebral amino acid imbalance [56, 58]. Because Tyr is a precursor of neurotransmitters in the brain's prefrontal cortex, Tyr deficiencies have been associated with cognitive dysfunction and ADHD in PKU patients [42, 59] and with decreased melanin synthesis, which contributes to light skin and hair [60]. Deficiencies of monoamine neurotransmitters (e.g., serotonin and norepinephrine) [61] in the brain of PKU patients have been frequently associated with some cognitive and neuropsychiatric symptoms [8, 62].

## Genetic etiology

### PAH gene associated with PKU

Pathogenic variants most often cause PKU in the *PAH* gene (OMIM 612,349) inherited in an autosomal recessive pattern. The *PAH* gene, mapped to chromosome 12q23.2, spans 90 kb and consists of 13 exons that are not equally distributed, as the exons are more condensed in the second moiety of the gene. The *PAH* gene's coding sequence is 1359 base pairs, which encode 452 amino acid polypeptides with a molecular weight of ~ 52 kDa [63]. The absence of TATA boxes characterizes the promoter region of the PAH gene. However, GC boxes, CCAAT boxes, CACCC boxes, two activator protein sites, partial glucocorticoid response elements, and partial cyclic AMP response elements are present [64]. The *PAH* gene is composed of three domains: an N-terminal regulatory domain (residues 1–142), a central catalytic domain (residues 143–410), and a C-terminal oligomerization domain (residues 411–452) with both a dimerization motif (residues 411–426) and a tetramerization motif (residues 427–452) [65].

Most variants were in the central domain (59.2%), followed by the N-terminal and the C-terminal of the PAH monomer (17.5% and 5.4%, respectively). The remaining variants (17.9%) were either intronic or UTR regions. Only 7.7% of all variants were in one of the four cofactor binding regions (the BIOPKU database). Of all variants, 58.1% were in-frame missense variants (58.1%), frameshift variants (13.9%), splicing variants (13.1%), nonsense variants (6.9%), and synonymous substitutions (4.9%) [1]. Out of approximately 1,186 variants known to date, exons 6 and 7 contained the largest number of variants (14.1% and 12.2%, respectively), followed by exon 3 (9.9%), and lesser frequencies in exons 11, 10, 12, 5 ranging from 9.3 to 4.9%. Thirty small insertions have been identified, mostly in intron 9 of the *PAH* gene (c.969 + 2insT) (http://www.biopku.org/home/home.asp).

### BH4-associated genes with HPA

Several reports have described BH4-related genes (for example, *GCH1 ‘GTPCH*,*’ PTS*, *PCBD1*, *QDPR*, and *SPR* genes) linked to HPA. The BH4 deficiency linked to HPA phenotypes is presented in BioDEF (http://www.biopku.org/home/biodef.asp) database as 11 in *GCH1*, 197 in *PTS*, 30 in *PCBD*, and 137 in *QDPR* genes, but no human SR deficiency without HPA [66]. To date, variants in *GCH1* with HPA clustered in exons 1, 5, and 6: one insertion (p.Pro9_Ala10insLeu), 8 missense (p.Glu56Lys, p.Leu92Ile, p.Arg184His, p.Met211Thr, p.Met211Ile, p.Met213Thr, p.Met213Ile, p.Arg235Gly), and 2 nonsense mutations (p.Gln110*, p.Glu242*). As for the *PTS*, few patients showed HPA alone but mixed phenotypes with HPA and neurotransmitter deficiency (e.g., p.Arg25Gly). However, some variants with HPA: p.Arg16Cys, p.Leu26Phe, p.Tyr113Cys, and p.Val124Leu are associated with a mild phenotypic HPA outcome [67, 68]. PTPS deficiency with HPA is relatively common in the Arab population, with c.238A > G (p.Met80Val) being the most common variant (allele frequency 33%) [69]. The p.Thr106Met variant accounts for 32% of all PTS alleles in Russia [70]. Thirty-two variants in the *PCBD1* gene have been found in HPA in the 5`-UTR, exons 2–4, and 7. Autosomal recessive DHPR deficiency responsible for the QDPR variants impairing the metabolism of serotonin (p.Gly151Ser and p.Phe212Cys) was associated with a very mild form of HPA (a novel homozygous splice site variant c.199-1G > T) [71]. Recently, Himmelreich et al. [72] have reviewed the insertion types in 800 cases with BH4 deficiency using the PNDdb (http://www.biopku.org/) and BioDEF (http://www.biopku.org/home/biodef.asp) as 5 in *GCH1*, 5 in *PTS*, 1 in *PCBD*, and 3 in *QDPR* genes.

### Large-scale deletion/duplication and inversion with PKU

Although its proportion may be underestimated, genomic large deletion and duplication (in a 12.9% and 2.1%, respectively) in the *PAH* gene [1] have been described in various ethnic populations [70, 73–82].

In addition, heterozygous and hemizygous inversions cause many different disorders, but severe phenotypes are persistent [83]. Unlikely, the chromosomal inversion has been rarely explored for the *PAH* and *BH4*-related genes, as the conventional cytogenetic analysis cannot allow the identification of exact breakpoints and thus discover the disruption of the chromosomal rearrangement. This issue may be resolved using PCR, Sanger sequencing, single-molecule real-time (SMART), and short-read genome sequencing approaches [84–86]. Lilleväli et al. [86] have detected a homozygous 9-Mb inversion between 4p16.1 and 4p15.32, disrupting the *QDPR* gene.

### Founder effects in PKU

Evidence supports founder variants within the *PAH* and BH4-related genes among various ethnic populations. For example, the literature indicates that single-exon deletions—EX3del4765, EX5del955, and the EX5del4232ins268—have founder effects of Slavic–Czech origin. These large deletions cause frameshifts and a loss of part of the *PAH* coding sequence, leading to no PAH activity [87]. In Yemenite Jewish, the 6.7 kb EX3del4765 deletion was seen as a founder variant [74, 76, 79]. The p.Phe20Leu and p.Glu81Glu in the *PTS* gene are reported to be specific to Filipino and Japanese populations [68, 88]. These variants were in linkage disequilibrium with a specific allele of the polymorphic microsatellite marker D11S1347, suggesting a founder effect for these frequent mutations [67, 88]. The haplotype analysis shows that Arg241Cys and Ex6-96A > G are exclusively associated with haplotype 4.3, suggesting founder effects in Taiwanese people [89]. However, local founder variants of R408Q, E286K and − 4173_− 407del, accounting for 21% of all mutant *PAH* alleles in Taiwan, are very rare or are undetected among PKU cohorts of other Asian regions [89]. In North Europe, PKU families in Southeastern Norway and Southern Iceland suggested nonsense Gly272* and Y377fsdelT founder variants [77, 90]. In Finnish ethnic populations, a negative founder effect could be diminished by the low incidence of PKU due to the extensive immigration and genetic drifts [91].

### Natural selection and pleiotropic effects of PKU

Pleiotropy can be described when one gene can influence two or more seemingly unrelated phenotypic traits. Gene pleiotropy occurs when a gene product interacts with multiple other proteins or catalyzes multiple reactions. Pleiotropic gene action can limit the rate of multivariate evolution when *natural selection*, sexual selection, or artificial selection on one trait favors one allele, while selection on other traits favors a different allele. Positive natural selection maintains or fixes beneficial variation and purifies (negative) selection that removes the deleterious genomic variations. Thus, genetic variants that predispose to disease could have been selected by natural selection if offering a survival advantage [92, 93]. In PKU, the *PAH*-responsible gene may be harmful and beneficial, referred to *antagonistic pleiotropy*. Several candidates have debated the antagonistic pleiotropic roles in neurodegenerative diseases (Huntington’s and Alzheimer’s), coronary heart disease, cystic fibrosis, and cancers, including PKU [94–98]. Earlier, the antagonistic pleiotropy was seen, whereas mothers of children with PKU had fewer miscarriages (i.e., potential benefit [98]) than controls who did not carry the gene [99], thus presenting positive selection [94].

### Protein–Protein interactions in PKU-responsible genes

**Fig. 3:**
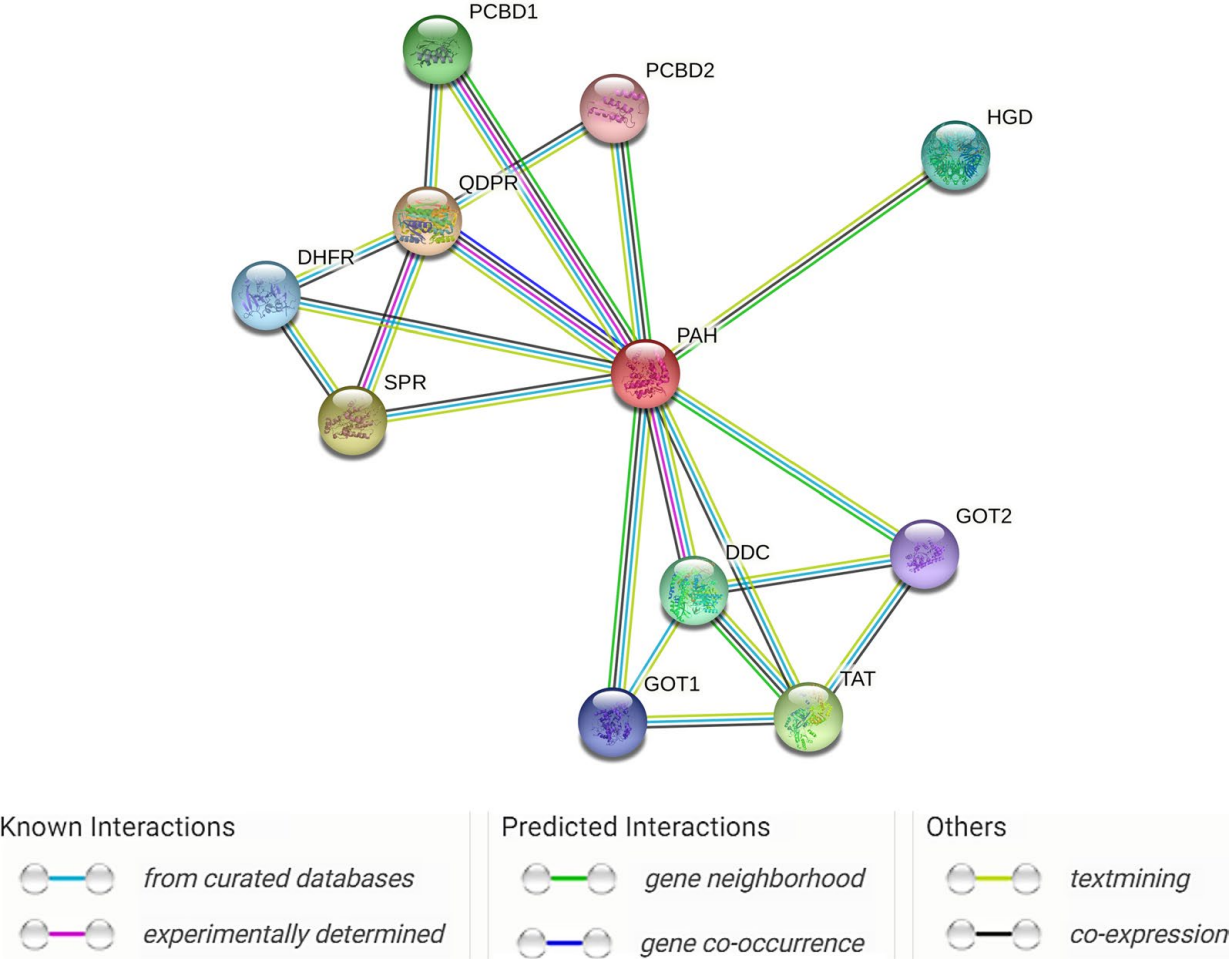
Protein network interactions contained the *PAH* and 10 related genes examined in this review created with STRING (https://string-db.org/), where there are strong interactions between the *PAH* gene and associated BH4 genes. Each node represents all the proteins (*n* = 11) produced by a single, protein-coding gene locus. Colored nodes describe proteins and the first shell of interactors. Edges represent protein–protein associations (*n* = 20) that are meant to be specific and meaningful, i.e., proteins jointly contribute to a shared function; this does not necessarily mean they are physically binding each other

We have used the Search Tool of the Retrieval of Interacting Genes (STRING) database (https://string-db.org) to predict functional interactions between proteins. Figure 3 presents the *PAH* protein network interactions with STRING software. The *PAH* protein network showed significantly more interactions among themselves (*P* value = 0.0038) than expected for a random set of proteins of the same size and degree of distribution drawn from the genome. Such an enrichment indicates that the proteins are partially biologically connected.

## Genotype–phenotype prediction system

PKU is a paradigm of a hereditary disease that can be treated via a low-Phe diet and BH4 (sapropterin HCl) treatment to prevent mental retardation and cognitive problems. Liemburg et al. [100] have recently reported that the Phe levels during the neonatal period are clearly and negatively related to later IQ. Although PKU is a monogenic disease, the genotype–phenotype correlation has no single explanation, whereas every patient has a unique complex PKU phenotype and will be treated accordingly [101, 102]. Thus, the Mendelian ‘simple’ PKU should be shifted toward a more complex disease phenotype [103]. Thus, several reports described inter-individual differences in the brain susceptibility to the toxic influence of HPA and the lack of intellectual disability in some untreated patients with PKU [104]. Modifier genes have been shown to contribute to regulating the Phe-related metabolic pathway [105]. However, the discovery of novel gene modifiers could contribute to better understanding of complex PKU phenotype and have therapeutic implications for the patients. Klaassen et al. [106] have reported that five SHANK variants could have protective modifying effect on the cognitive development of PKU patients escaping intellectual disability. Moreover, Bik-Multanowski et al. [107] have reported that the common SLC7A5 rs113883650 variant could affect the Phe level in the brain.

Splicing, nonsense, and frameshift variants in the *PAH* gene cause loss of function (i.e., are null variants), whereas missense variants and some in-frame insertions and deletions (indels) cause defective protein translation [108]. Identifying genotypes and associating them with patients' phenotypes could significantly improve the treatment of patients [108]. Correlations between many genotypes and patient phenotypes are already available in the literature and public databases (http://www.biopku.org). Genotyping can provide information on how *PAH* variants occur and the effect of these variants on PAH enzyme function. Genotyping can also provide information on a patient's potential responsiveness to BH4 treatment, enhancing PAH activity by conferring a chaperone-like effect on the enzyme's misfolded subunit [109].

Compound heterozygotes can result in more than 2,600 known causing genotypes. Variants have been assigned to phenotype categories of classic PKU, moderate PKU, mild PKU, and HPA, with the genotype–phenotype predictions formulated as arbitrary values (AVs) [110]. Classic PKU variants are assigned an AV of 1 (nearly no PAH activity for nonsense variants, splicing variants, indels, and < 10% of wild-type PAH activity for missense mutations). Moderate PKU variants are assigned an AV of 2 (10–30% of wild-type PAH activity), mild PKU variants are assigned an AV of 4 (30–70% of wild-type PAH activity), and mild HPA variants are assigned an AV of 8 (> 70% of wild-type PAH activity) [110].

In brief, a phenotype resulting from two variant alleles is expressed as the sum of the AVs of the two variants. According to Guldberg et al. [110], this calculation was since the milder variant (with a higher APV) is always dominant over the severe one [15]. Some disagreements with the AV estimation [111–113] would arise as possible effects of interallelic complementation and epigenetic factors, which may influence the phenotype [101, 114].

## Screening and diagnosis

### Newborn screening

Newborn screening programs for PKU have been implemented worldwide to check Phe levels in neonates. The screening involves a heel prick to collect blood drops, spotted onto filter paper (i.e., a Guthrie card) and dried [17]. The bacterial inhibitory assay (BIA) is semiquantitative with limited sensitivity, so false-negative results are possible [17]. More accuracy has been achieved with a fluorimetric microassay (FMA) using chromatographic separation technology. In contrast to both the BIA and the FMA, specific for Phe, tandem mass spectrometry (TMS) can quantitatively measure all amino acids, including Phe and Phe-Tyr ratios, with a low false-positive rate and excellent accuracy and precision [45, 55].

Regarding diagnosis of HPA, assays need to be able to differentiate between PAH deficiency, BH4 disorders, and *DNAJC12* defects. Dihydrobiopterin reductase (DHPR) activity can be measured in a DBS or a urine sample, though accuracy is higher from a DBS. In brief, 20 mg/kg of sapropterin dihydrochloride is taken orally, and Phe concentration is measured in a DBS before loading and at 4, 8, 16 and 24 h after loading. Patients with BH4 defects and genetic variants in *DNAJC12* show a considerable decrease in Phe blood concentration up to 8 h after BH4 administration, but patients with BH4-responsive PKU or DHPR deficiency tend to show a much slower decrease in blood Phe. If no decrease in Phe occurs, the patient probably has PAH deficiency, although such a result cannot be used to conclude that a patient has non-BH4-responsive PKU, as some neonates with a negative BH4 loading test are BH4-responsive when tested at an older age [115].

BH4 synthesis and regeneration is a multi-step process catalyzed by five enzymes. All the BH4 disorders are inherited in an autosomal recessive manner, apart from GTPCH enzyme deficiency, which manifests with both autosomal recessive and autosomal dominant inheritance patterns (Table 1). Prenatal diagnosis of BH4 deficiency is possible by evaluating the pterin metabolites biopterin and neopterin concentrations in amniotic fluid. The pattern of these metabolites in amniotic fluid reflects the pattern seen in the urine of the same patients after birth and is therefore diagnostic for enzyme deficiencies of GTPCH (MIM 600,225) and PTPS (MIM 612,719). However, molecular analysis is now the method for diagnosing all primary BH4 deficiencies [64].

**Table 1:** Nomenclature for BH4 disorders

Disease name	Gene symbol	Inheritance	Affected enzyme	OMIM	Gene locus
GTP cyclohydrolase 1 deficiency	*GCH1*	AD	GTPCH1	128,230	14q22.2
GTP cyclohydrolase 1 deficiency	*GCH1*	AR	GTPCH1	233,910	14q22.2
6-pyruvoyl-tetrahydropterin synthase deficiency	*PTS*	AR	PTPS	261,640	11q23.1
Sepiapterin reductase deficiency	*SPR*	AR	SR	612,716	2p13.2
Q-dihydropteridine reductase deficiency	*QDPR*	AR	DHPR	261,630	4p15.32
Pterin-4-alpha-carbinolamine dehydratase deficiency	*PCBD1*	AR	PCD	264,070	10q22.1

*AD* Autosomal dominant, *AR* Autosomal recessive, *BH4* Tetrahydrobiopterin, *GTP* Guanine triphosphate

## Molecular diagnosis

Two of the first variants identified in the *PAH* gene were c.1315 + 1G > A and c.1222C > T (p.Arg408Trp) [79, 97]. Within a few years, many new variants were identified, and the high variability of the *PAH* gene became evident. These variants were added to screening protocols, but they were detected using radioactive isotopes, making the process time-consuming and laborious or applying the PCR-dependent DGGE apparatus [116]. The development of Sanger sequencing in 1977 presents the gold standard for gene variant detection in PKU patients. This direct approach is suitable for sequencing hot spot point mutations or small genes [117], but it is rather costly and time-consuming for large or multiple genes, e.g., genes associated with PKU and BH4-deficiency. Next-generation sequencing (NGS) is a faster and inexpensive technology allowing massive parallel deep-level sequencing. NGS enables simultaneous analysis of many samples for a couple of genes to the whole genome [64, 106, 118]. Various ethnic studies utilized reliable NGS technologies to update the mutational spectrum of PKU among their populations [119, 120]. It is likely that with the reduction in cost and wide application of NGS, newborn genetic screening has recently received more attention [121–125]. Multiple studies have assessed the effect of HPA on liver functions in PKU in mice using transcriptome and proteomic analyses [126].

## Management of PKU

### Clinical manifestations

If PKU is untreated, patients can experience severe intellectual disability, epilepsy, seizures, psychiatric movement behaviors, microcephaly, generalized hypopigmentation of skin (including eyes and hair), eczema, and a musty sweat odor [7]. However, with early intervention after birth, dietary treatment can prevent sequelae. Late diagnosed or untreated PKU may be due to newborn screening failures and is most common in countries without newborn screening protocols or treatment [127, 128]. If treatment is not adequate, clinical signs can include lower extremity spasticity and cerebellar ataxia, tremor, encephalopathy, and visual abnormalities [129, 130]. Some cases may not be diagnosed until adulthood, presenting with mild-to-moderate neurological complications related to PKU [131, 132]. Since brain damage is one of the greatest risks for PKU patients, early detection and assessment of neural activity are important for patient health. Sometimes, dementia may be associated with PKU in adulthood [132]. However, treatment helps prevent major neurological deficits, cognitive abnormalities, and specific learning disabilities immediately after birth.

PAH-deficient individuals have normal biopterin content in blood and urine, but oral administration of additional BH4 to some individuals with mild HPA significantly reduces blood Phe levels without altering dietary Phe content [133–137]. In a few case reports, untreated individuals with mild PAH deficiency and normal intelligence were diagnosed in adulthood due to sudden and severe psychiatric deterioration. The possibility of BH4 deficiency should be investigated in all infants with milder forms of HPA [55, 138].

### Phenylalanine-restricted diet

Dietary control of PKU is challenging but possible. As Phe is an essential amino acid, patients with PKU must use a diet containing low-Phe concentrations to maintain blood Phe at 2–6 mg/dL (120–360 μmol/L) throughout the life span as recommended by the US National Institutes of Health. The European and US guidelines recommend treating individuals with PKU when Phe levels exceed 360 mmol/L [12, 45, 139]. Long-standing dietary deficiency in protein leads to a decrease in vitamin B12 (found in meat, poultry, and fish), as well as a decrease in calcium and vitamin D. Thus, supplements rich in minerals and vitamins must be taken to avoid growth retardation and osteoporosis [55, 140, 141].

The dietary treatment comprises three aspects: restricting natural protein intake, supplementing with a low-Phe or Phe-free amino acid mixture, and consuming low-protein food products. Phe restriction can only be performed by restricting the intake of natural protein. The extent of natural protein (Phe) restriction is based on the amount of Phe required for net protein synthesis (e.g., age-dependent growth and balance between anabolism and catabolism in periods of illness) and the severity of the PAH deficiency [139, 140]. During restricted Phe consumption, the intake of other essential amino acids, vitamins, minerals, and carnitine should be balanced. However, natural protein can be replaced with an amino acid mixture that lacks Phe but is enriched in Tyr. Moreover, intake of low-protein foods containing carbohydrates and fats may replace basic foods such as bread and pasta to supply energy [142]. Enormous improvements in intellectual and cognitive outcomes have been observed in PKU patients when dietary Phe is restricted before considerable damage has occurred [124].

A diet that includes glycomacropeptides (GMP), a protein component of whey that is completely lacking Phe, has been reported to taste better, increase the feeling of satiety, and improve immunological aspects of PKU by decreasing inflammation [143–146]. Daly et al. [146] have presented evidence that casein GMP supplemented with amino acids (GMP-AA) also results in less variability in blood Phe levels than a Phe-free diet [146]. However, due to the weak absorption of GMP medical foods [147], ethylcellulose and alginates are added to this Phe-free protein substitute [142].

### Maternal PKU

Poor treatment of PKU in pregnant women (known as maternal PKU) may increase the risk of abnormal fetal developmental and a fetal teratogenic effect [148]. This effect occurs especially within the first 8–10 weeks of gestation regardless of the genetic PKU status of the fetus. The teratogenic effects can occur in intrauterine growth restriction, postnatal growth and psychomotor retardation, microcephaly, and congenital heart defects [149, 150]. These effects could be prevented by maternal diet control before and throughout pregnancy with a plasma Phe concentration of 120–360 μmol/L or a Phe level < 240 μmol/L [140] reported a 14% chance for offspring to develop chronic heart disease (CHD) if the mother's basal Phe concentration was 900 mmol/L or higher, compared with only 2% if it was < 900 mmol/L [140]. Also, the type of PKU did not correlate with CHD development, as the basal Phe level in some women with mild PKU was < 1,500 mmol/L, and the risk increased if metabolic control did not start as early as the eighth week of gestation [151]. The mechanism of maternal PKU is unknown, but the placenta's ability to concentrate Phe on the fetal side may be a major factor. Although the fetus may be heterozygous for a PKU variant, the immature hepatic enzyme system of the fetus may be the reason for low transplacental Phe uptake. Treatment with sapropterin has been slowly introduced to manage Phe concentrations in women with BH4-responsive PKU who aim to become or are pregnant, and it leads to increased tolerance for Phe and no excess fetal abnormalities [152, 153]. However, a registry of fetal abnormalities in women with maternal PKU treated by diet or BH4 is lacking. Growth of the placenta in weeks 16–22 of gestation requires a diet that restricts Phe intake, putting the fetus at risk of hypophenylalaninemia [154]. Thus, maternal PKU management should also include total protein, supplements with low Tyr, and folic acid supplementation to minimize this risk.

### Amino acid supplementation

LNAA supplements can be administered, especially to adult patients with PKU in whom a Phe-restricted diet is not tolerable [155]. LNAAs include the branched-chain amino acids (valine, leucine, and isoleucine) and the aromatic amino acids (tyrosine, tryptophan, threonine, methionine, and histidine) [156, 157]. These supplements compete to bind the LNAA transporters based on their plasma concentrations. High levels of LNAA supplementation inhibit plasma Phe transport and reduce the amount of Phe that crosses the blood–brain barrier [158]. However, it should not be used in women of childbearing age [159].

### Sapropterin and pegvaliase therapy

**Fig. 4:**
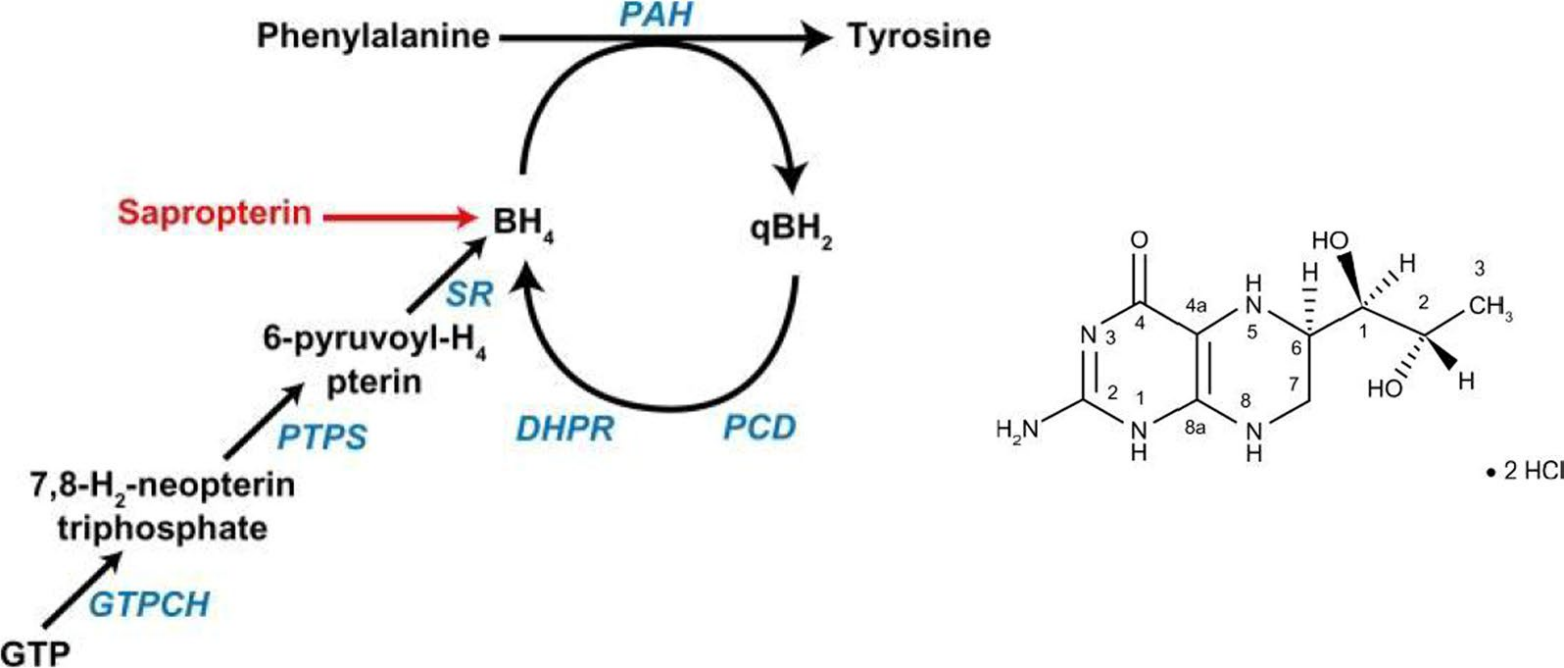
Role of sapropterin as a synthetic form of BH4. Fully active BH4 is regenerated through the sequential action of pterin-4a-carbinolamine dehydratase and dihydropteridine reductase (DHPR) or may be synthesized de novo from guanosine triphosphate (GTP) [50]

The BH4 synthetic analog sapropterin dihydrochloride (Kuvan®, BioMarin Corporation, Tiburon, CA), approved by FDA, is indicated for the treatment of HPA in patients with PKU or BH4 deficiency [160]; consequently, some patients with PAH deficiency can now skip dietary treatment [161]. PAH hydroxylates Phe through an oxidative reaction with BH4 as a cofactor to form tyrosine. Sapropterin therapy creates an excess of the cofactor (Fig. 4), which activates residual PAH enzyme, improving Phe metabolism and decreasing Phe concentrations in the blood of PKU patients. PKU patients treated with sapropterin must continue their restricted Phe diet and undergo regular clinical assessment and monitoring of blood Phe and Tyr concentrations, nutrient intake, and psychomotor development [162]. Because of this complexity, sapropterin therapy is difficult to maintain throughout life, and dietary noncompliance is common. Furthermore, hypophenylalaninaemia is a direct adverse effect of sapropterin therapy that, if it occurs, requires dose adjustment or increased dietary Phe intake. In clinical trials, other common adverse events among patients treated with sapropterin have included headache, rhinorrhoea, pharyngolaryngeal pain, vomiting, diarrhea, nasal congestion, and cough [163].

Most individuals with mild or moderate PKU may be responsive to sapropterin, while up to 10% of individuals with classic PKU respond to a pharmacologic dose of BH4 (10–20 mg/kg per day) [164]. In a study in a Croatian population, Phe levels decreased by more than 50% 8 h after sapropterin treatment in all patients with mild HPA and more than half of patients with mild PKU; however, only 8% of patients with classic PKU responded [165]. Moreover, Klaassen et al. [166] reported an insignificant increase in PAH levels with sapropterin treatment in HPA cases who had the p.Gln226Lys variant in the *PAH* gene. This could illustrate the significance of genotype information for achieving more personalized medicine.

### Pegvaliase therapy

The new therapeutic pegvaliase (PALYNZIQ®, BioMarin Pharmaceutical Inc., USA) is a novel enzyme substitution therapy for PKU that the FDA approved in 2018 for adults in the USA and for patients ≥ 16 years of age in Europe who have uncontrolled blood Phe concentrations > 600 μmol/L. Pegvaliase is an enzyme substitution therapy using PEGylated recombinant *Anabaena variabilis* PAL to lower blood Phe levels to normal ranges irrespective of residual PAH [167–169]. Pegvaliase is the first therapeutic option that has the potential to lower blood Phe levels to normal ranges irrespective of BH4 or genotyping data [167, 167]. Despite its effectiveness, adverse side effects include skin reactions, arthralgia, and rare anaphylactic responses [170].

### Gene therapy

Gene therapy is generally concerned with modifying genes in cells to produce a therapeutic effect or treating disease by repairing or reconstructing defective genetic material [171]. Decades ago, the American geneticist Martin J. Cline (1980) was the first to try modifying human DNA, but the first method for nuclear gene transfer in humans was achieved by scientists at the National Institutes of Health (NIH, 1989). Later, William F. Anderson performed the first therapeutic gene transfer and direct insertion of human DNA into the nuclear genome to cure several genetic disorders [172]. The US Food and Drug Administration (FDA) approved several drug therapies utilizing adeno-associated viruses (AAVs) and lentiviruses to introduce a copy of a gene with no deleterious mutations and thus produce a functional protein by *in vivo* and *ex vivo* transfer. This gene replacement therapy has successfully treated several recessive and inherited dominant disorders [173].

Genetically engineered knockout mice are mice in which a particular gene is inactivated (i.e., knocked out) by replacing it with a modified mutated version or introducing an artificial DNA fragment that disrupts the target gene's function. These methods, known as gene targeting methods, demand intense labor for the use of embryonic stem cells due to their ability to differentiate into nearly any adult cell type. CRISPR-Cas9, a DNA sequence derived from DNA fragments of bacteriophages previously infected by the bacteria and associated with a Cas9 nuclease RNA-guided system, can recognize and cut at the desired location into the cell's genome. This allows existing genes to be removed and/or new ones added in vivo [174]. However, knock-in mutations, facilitated by homology-directed repair (HDR), are the traditional pathway of targeted genomic editing approaches. Thus, CRISPR-Cas9 provides sufficient PAH activity (> 20% of normal) in Pah^enu2^ mice to restore physiological blood Phe concentrations [175]. Singh et al. [176] have recently created a homozygous Pah knockout mouse model (known as Hom-mice) using CRISPR/Cas9 to change the GAG (codon 7) in the *PAH* gene to a stop codon TAG. The physiological features of the mice included higher levels of Phe in the blood and brain, retarded body growth, hypopigmentation, lower myelin content, and lower levels of Tyr in the brain [176].

Pan et al. [177] have tried to correct the most common variant c.1222C > T (p.Arg408Trp) in the *PAH* gene in COS-7 cells as in vitro model by CRISPR-*FokI* nuclease RNA guide complex. The results indicated the correction of the targeted nucleotide, and thus the PAH activity increased with the RS-1 enhancer inclusion [178].

The Pah^enu2^ mouse, the most widely studied animal model, generated by ethylnitrosourea (enu)-induced random mutagenesis, can be used for investigating the pathophysiological mechanisms underlying PAH deficiency (Table 2). The BTBR.Cg-Pah^enu1^ mouse with a PAH p.Val106Ala mutation [178] is used as a model of BH4-responsive PKU in humans [179], which can be treated with the BH4 synthetic analog sapropterin dihydrochloride [180]. This mouse model was also generated by ENU-induced random mutagenesis, and its severe BH4-non-responsive phenotype is a model of untreated or late-treated severe PKU in humans [181].

**Table 2:** Managing therapies for PKU

Therapy	Delivery	Physiological mechanism	Dose
Gene correction	Systemic	Delivery of base-editing to correct variants in the *PAH* gene	One IV
Gene therapy	Systemic	HMI-102: provision of the normal *PAH* cDNA to hepatocytes (AAV, lentivirus or naked DNA)	One IV
mRNA therapy	Systemic	Provision of lipid nanoparticle-encapsulated PAH mRNA	IV, SQ; frequency TBD
Enzyme substitution	Systemic	RTX-134: Anabaena variabilis PAL expressed in universal @donor red blood cells	IV; frequency TBD
	Oral	SYNB1618: bacteria overexpressing PAL to metabolize Phe in the gut	Oral; three times daily
	Oral	Oral CDX-6114: PAL genetically modified to retain activity after oral administration to metabolize Phe in the gut	Oral; three times daily
Cofactor therapy	Oral	Oral CNSA-001: sepiapterin, a precursor of tetrahydrobiopterin, to stimulate residual enzyme activity of mutant PAH	Oral; once daily

*AAV* Adeno-associated virus, *cDNA* Complementary DNA, *IV* Intravenous, *PAH* Phenylalanine hydroxylase, *PAL* Phenylalanine ammonia-lyase, *Phe* Phenylalanine, *SQ* Subcutaneous, *TBD* To be determined [1]

Another approach studied by an American team is utilizing gut microbes as a manufacturer producing nutrients or degrading toxic products within the host physiology. Isabella et al. [182] utilized *E. coli Nissle* to synthesize the SYNB1618 to treat PKU in a *Pah*^*enu2/enu2*^ mouse model and healthy Cynomolgus monkeys. The SYNB1618 considered two pathways to be targeted to lower the Phe within the host, utilizing phenylalanine ammonia-lyase (PAL) that converts Phe into *trans*-cinnamate (tCA), in addition to L-amino acid deaminase (LAAD), which convert Phe to phenylpyruvate [182]. Both pathways require the addition of a high-affinity Phe transporter that was added to the plasmid vector [182].

## Conclusion

This review debates the epidemiology, pathophysiology, genetic etiology, and management of PKU. It highlights the founder effects and the potential benefit of antagonistic pleiotropy in the disease. The PAH enzyme, expressed predominantly in the liver (and kidney and pancreas), is responsible for the converting Phe to Tyr in the presence of the co-substrate tetrahydrobiopterin (BH4). Here, some interesting points should be referred to: (1) In some cases, the genotype–phenotype prediction system of this monogenic PKU exhibits more complex phenotypes than expected, for example, lack of intellectual disability in some untreated PKU patients and inter-individual differences in the brain susceptibility to the toxic influence of HPA. (2) Although PKU is a paradigm of a hereditary disease that can be treated via a low-Phe diet and BH4 (sapropterin-HCl) treatment to prevent mental retardation and cognitive problems, the discovery of novel candidate PKU modifiers should be investigated to have therapeutic implications for the patients. (3) Despite several remaining obstacles, the rapid, reliable, inexpensive NGS should likely enter many newborn screening programs because of its ability to simultaneously analyze many samples for a couple of genes to the whole genome, and thus better diagnose conditions and offer personalized treatments.

## Data Availability

The data sets analyzed during the current study are available from the corresponding author.

## Abbreviations

AAVs: Adeno-associated viruses
ADHD: Attention-deficit hyperactivity disorder
Avs: Arbitrary values
BH2: Dihydrobiopterin
BH4: Tetrahydrobiopterin
BIA: Bacterial inhibitory assay
CHD: Chronic heart disease
DBS: Dried blood spot
DGGE: Denaturing gradient gel electrophoresis
DHPR: Dihydropteridine reductase
*enu*: Ethylnitrosourea
FDA: Food and Drug Administration
GMP: Glycomacropeptide
GTP: Guanosine triphosphate
Hom-mice: Pah knockout mouse model
HPA: Hyperphenylalaninemia
LNAA: Large neutral amino acid
PAH: Phenylalanine hydroxylase
PAL: Phenylalanine ammonia-lyase
Phe: Phenylalanine
PKU: Phenylketonuria
qBH2: Quinonoid dihydrobiopterin
tCA: *trans*-Cinnamate
TMS: Tandem mass spectrometry
Tyr: Tyrosine
